# Rational Design of Nucleoside–Bile Acid Conjugates Incorporating a Triazole Moiety for Anticancer Evaluation and SAR Exploration

**DOI:** 10.3390/molecules22101710

**Published:** 2017-10-12

**Authors:** Maria Luisa Navacchia, Elena Marchesi, Lara Mari, Nicola Chinaglia, Eleonora Gallerani, Riccardo Gavioli, Massimo Luigi Capobianco, Daniela Perrone

**Affiliations:** 1 Consiglio Nazionale delle Ricerche, Istituto per la Sintesi Organica e la Fotoreattività (CNR-ISOF), via P. Gobetti 101, 40129 Bologna, Italy; massimo.capobianco@isof.cnr.it; 2 Dipartimento di Scienze Chimiche e Farmaceutiche, Università degli studi di Ferrara, via L. Borsari 46, 44121 Ferrara, Italy; mrclne@unife.it (E.M.); mralra@unife.it (L.M.); nicola.chinaglia@unife.it (N.C.); prd@unife.it (D.P.); 3 Dipartimento di Scienze della Vita e Biotecnologie, Università degli studi di Ferrara, via L. Borsari 46, 44121 Ferrara, Italy; eleonora.gallerani@unife.it (E.G.), riccardo.gavioli@unife.it (R.G.)

**Keywords:** bioconjugates, bile acids, nucleosides, click chemistry, cytoselectivity, anticancer activity

## Abstract

Herein we report a study on the synthesis and biological evaluation of a library of nucleoside-bile acid conjugates prepared by combining 2′-deoxyadenosine, 2′-deoxyguanosine, 2′-deoxyuridine as well as adenosine and guanosine derivatives with cheno-, urso-, *nor*-cheno-, *nor*-urso- and taurourso-desoxycholic acid derivatives by means of the click reaction. The new nucleoside-bile acid conjugates incorporating a triazole moiety were tested in vitro against leukemic K562 and HCT116 colon carcinoma, as well as on normal fibroblast cells. Six compounds displayed interesting anti-proliferative activity against the selected cancer lines and no cytotoxic effects against normal fibroblasts. A possible structure activity relationship was also investigated.

## 1. Introduction

Important traditional chemotherapeutic drugs or anticancer agents were mostly derived from natural sources through synthetic structural modifications. Successful examples of this approach are represented, among others, by the anthracyclines, taxanes and camptothecins that are still considered a structural platform for discovering new anticancer drugs [1].

Nucleosides and nucleotides—endogenous small molecules that can be chemically fine-tuned leading to the corresponding analogues—can behave as antimetabolites and can inhibit the cellular division and viral replication by incorporation into DNA or RNA, resulting in potential therapeutic benefits. They can also act as inhibitors of essential enzymes such as DNA polymerases, kinases and so on. In such a way, they would operate by stopping the synthesis of pre-DNA molecule building blocks or by direct damage of the DNA in the nucleus of the cell or by effecting the synthesis or by breakdown of the mitotic spindles. Currently, several nucleoside and nucleotide analogues derived from 2′-deoxycitidine, 2′-deoxyadenosine and 2′-deoxyguanosine have been approved by the FDA as anti-cancer drugs or anti-viral agents [2].

Despite their therapeutic potential, the bioavailability of hydrophilic nucleoside-based drugs remains a critical negative feature since they do not readily cross the plasma membrane by passive diffusion, and accordingly, their clinical efficacy also depends on nucleoside delivery systems [3,4].

With the aim to discover new nucleoside analogues with anticancer activity we consider conjugation as a powerful approach. In principle, a targeted conjugation can be helpful to tune the cytotoxicity, for instance by coupling a 2′-deoxyadenosine derivative with a NO photodonor unit the intrinsic cytotoxicity of the bioconjugate combined upon light irradiation with that of the photogenerated NO leads to an interplay of anticancer mechanisms of action [5]. Furthermore, 2′-deoxyadenosine derivatives conjugated with cheno- and urso-deoxycholic acids through a triazole or a thioalkyl unit tested on four cancer cell lines (K562, Jurkat, HCT116 and A2780) showed interesting antiproliferative activity selectively towards leukemic T-cells whereas no cytotoxicity against the solid tumors HCT116 and A2780 was found [6]. In our studies, bile acids (BAs) were chosen as combination partners by virtue of their biological as well as physico-chemical properties. For instance, the cytotoxic activity of certain BAs and BA-derivatives is well recognized, including the potential of several unconjugated BAs to induce cell death in a wide range of cells, through their non-specific ability to disrupt cell membranes (biological surfactant feature) or receptor-mediated interactions and DNA oxidative damage [7]. On the other hand, the conjugation with hydrophilic glycine and taurine can dramatically decreases BA cytotoxicity while enhancing the neuroprotective effects [8,9]. Moreover, taking advantage of their organotropism in the enterohepatic circulation mediated by the BA transport systems, the presence of BA units can be helpful in targeting a drug conjugate to the liver or to improve its metabolic stability [10]. It has been also reported that the conjugation of zidovuidine (AZT), a nucleoside analogue-based drug, with ursodesoxycholic acid increases the poor permeability of AZT through the intracellular departments [11]. Thanks to their intrinsic chemical features BAs can be fine tailored.

We present herein a study on the synthesis and biological evaluation of nucleoside-BA conjugates obtained by combining a selection of nucleoside analogues and bile acid derivatives. For this purpose, 2′-deoxyadenosine (dA), 2′-deoxyguanosine (dG), 2′-deoxyuridine (dU) as well as adenosine (A) and guanosine (G) modified at a suitable position with an alkynyl chain containing an acetylenic bond were conjugated by means of the click reaction with cheno- (CDC), urso- (UDC), taurourso- (TUDCA), *nor*-cheno (*nor*-CDC) and *nor*-urso- (*nor*-UDC) deoxycholic acid derivatives equipped with the azido group at the head or the tail position. The new nucleoside-BA conjugates were characterized and tested in vitro against two types of cancer cell lines: leukemic K562, a hematological cancer, and the solid tumor HCT116 colon carcinoma, as well as on normal fibroblast cells.

## 2. Results

The click chemistry approach, being a specific and high yield reaction, was considered a good synthetic approach for the preparation of our target nucleoside-BA hybrids. Moreover, the triazole moiety resulting from the 1,3-cycloaddition is biologically relevant, being able to improve the biostability, bioavailability and also the anticancer activity of bioactive compounds [12,13,14,15].

**Scheme 1:**
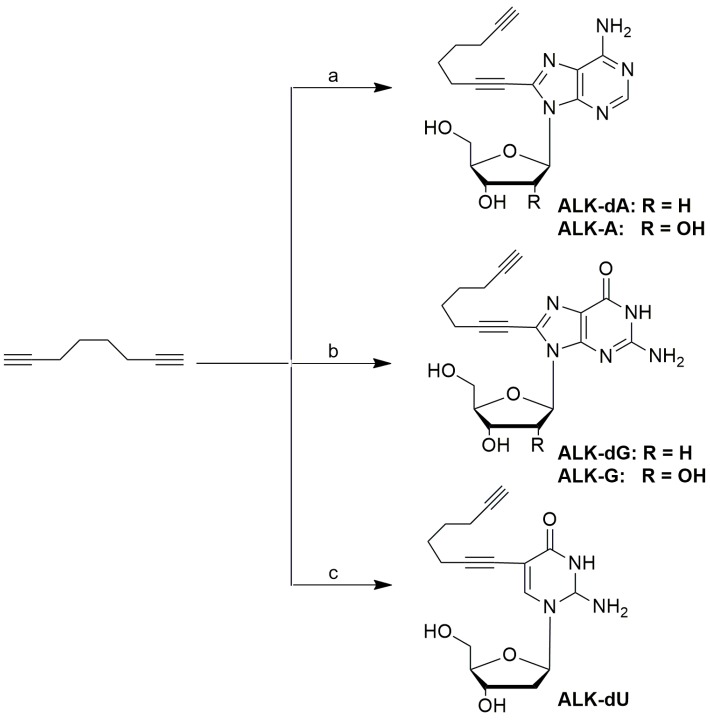
Synthesis of the alkyne-nucleoside intermediates. *Reagents and conditions*: (PPh_3_)_2_PdCl_2_, CuI, DMF, TEA, 50 °C, 2.30 h; (**a**): 8-Br-2′-deoxyadenosine or 8-Br-adenosine; (**b**): 8-Br-2′-deoxyguanosine or 8-Br-guanosine; (**c**): 6-I-2′-deoxyuridine.

Click chemistry requires the presence of a terminal alkyne moiety and an azido group. To provide those features the nucleoside units were modified with an alkynyl moiety at C-8 position in the case of the purine bases and at the C-6 position in the case of the pyrimidine one in order to keep unchanged their intrinsic characteristic of recognition of natural nucleic acids through specific hydrogen bond patterns (Watson-Crick and Hoogsteen). 8-(1,7-Octadynyl)-2′-deoxyadenosine (**ALK-dA**) was prepared as previously reported [6]. Similarly, the 8-(1,7-octadynyl)-derivative of A, dG and G, as well as 6-(1,7-octadynyl)-2′-deoxyuridine (namely **ALK-A**, **ALK-dG**, **ALK-G** and **ALK-dU**, respectively) were synthesized through a standard palladium catalyzed cross-coupling reaction starting from commercially available 8-bromo-A, 8-bromo-dG, 8-bromo-G and 6-iodo-dU [16] (Scheme 1).

**Scheme 2:**
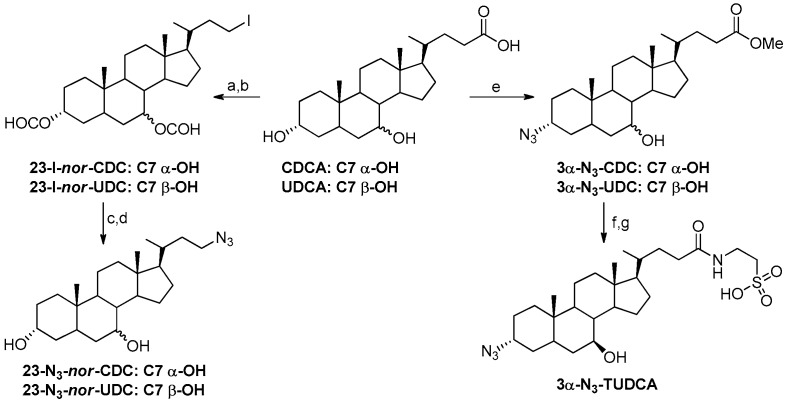
Synthesis of BA-azide intermediates. *Reagents and conditions*: (**a**): HCOOH, 55 °C, 24 h; (**b**): DIH, DCE, hν, reflux 2 h; (**c**): NaN_3_, DMF, r.t., 6 h; (**d**): NaOH 25%, MeOH, r.t., 2 h; (**e**): see reference [6]; (**f**): 1.5 M LiOH in MeOH, r.t., 21 h; (**g**): TEA, ClCOOEt, THF, 0 °C to r.t., then taurine, NaOH 10%, r.t., 12 h.

The azido-BA derivatives **3α-N_3_-CDC** and **3α-N_3_-UDC** were synthesized starting from commercially available BAs, using a synthetic approach that we described previously [6,17] (Scheme 2). The 3α-N_3_ derivative of TUDCA was prepared in three steps in 75% overall yield from the corresponding **3α-N_3_-UDC**: the methyl ester was hydrolyzed with a 1.5 M LiOH in MeOH to the corresponding acid, which in turn was coupled with the aminoethanesulfonic acid taurine after activation of the free acid with ethyl chloroformate (Scheme 2).

Finally, the **23-N_3_-*nor*-CDC** and **23-N_3_-*nor*-UDC** were synthesized starting from the corresponding bile acids following a recently reported metal free iodo-decarboxylation method [18]. Accordingly, the C3 and C7 free hydroxyl groups of CDCA and UDCA were firstly protected as formyloxy derivatives by using formic acid at 55 °C for 24 h, then concentrated in vacuo and the residues irradiated for 2 h in presence of 1,3-diiodo-5,5-dimethylhydantoin (DIH) as a sole reagent. After purification by flash chromatography the **23-I-*nor*-CDC** and **-UDC** derivatives (85–90% yield) were converted into the target compounds through a nucleophilic substitution with sodium azide in DMF at room temperature, followed by hydrolysis of the formate esters with 25% NaOH which allowed the precipitation of **23-N_3_-*nor*** BAs derivatives as pure compounds in satisfactory yields (75–78% after two steps; Scheme 2).

**Scheme 3:**
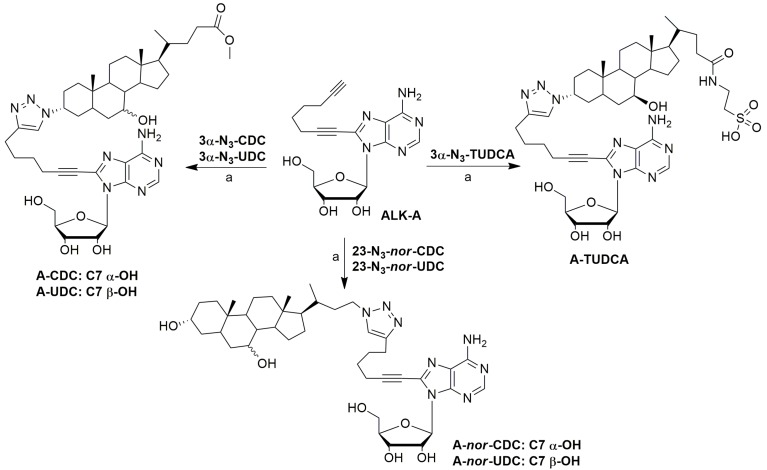
Synthetic scheme for the preparation of **A-CDC**, **A-UDC**, **A-*nor*-CDC**, **A-*nor*-UDC** and **A-TUDCA** conjugates. *Reagents and conditions*: (**a**): CuSO_4_·5H_2_O, sodium ascorbate, 1:1:1.5 H_2_O/*tert*-BuOH /THF (*v*/*v*), 25 °C, 18 h.

The click chemistry was performed via a Cu (I)-mediated 1,3-dipolar cycloaddition reaction under commonly used conditions: a (1:1:1.5) H_2_O/*tert*-BuOH/THF (*v*/*v*) solution of the appropriate alkyne–nucleoside derivative and of the BA-azide in the presence of the CuSO_4_ catalyst and sodium ascorbate was stirred at room temperature for 18 h. The target conjugates were obtained in yields ranging from 60% to 90% after purification. In Scheme 3 is depicted for example, the synthesis of the conjugate compounds of adenosine with all the BA selected (namely **A-CDC**, **A-UDC**, **A-TUDCA**, **A-*nor*-CDC** and **A-*nor*-UDC**).

**Figure 1:**
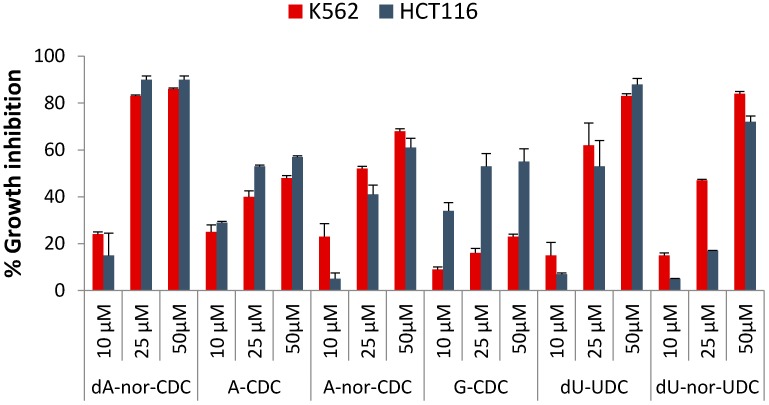
Antiproliferative activity of the most active conjugates against K562 and HCT116 cancer cells at 10, 25, 50 μM after 72 h.

All conjugated compounds prepared via click chemistry and listed in Table 1 were evaluated in vitro for their cytotoxic activity against K562 leukemia cells and the colon cancer cell line HCT116. Normal human skin fibroblast cells were chosen as a control and cisplatin served as a reference compound. The cytotoxicity was evaluated using the MTT assay (details are reported in the Materials and Methods section). In all experiments the cell growth inhibition of K562 and HCT116 was determined for each compound at concentrations of 10, 25 and 50 μM after 72 h of treatment and up to 100 μM in the case of fibroblasts. Figure 1 shows the antiproliferative activity of the most active conjugates against K562 and HCT116 cancer cells whereas in Table 1 the IC_50_ values are reported for all the compounds tested, including the alkyne-nucleoside and BA-azide building blocks.

With regard to the 2′-deoxyadenosine derivatives, **dA-*nor*-CDC** was found cytotoxic against both K562 and HCT116, with comparable IC_50_ values (16.2 and 17.0 μM, respectively). On the other hand, **dA-*nor*-UDC** showed a preferential cytotoxicity against HCT116 cancer cells (IC_50_ = 44.8 μM vs. 87.1 μM extrapolated value; Table 1).

In the case of the adenosine derivatives, both **A-CDC** and **A-*nor*-CDC** were found active against K562 and HCT116 but with an opposite cytoselectivity. Indeed, **A-CDC** showed a higher cytotoxicity against HCT116, whereas **A-*nor*-CDC** was found more toxic towards K562. None of the bioconjugates of 2′-deoxyguanosine and guanosine series showed any cytotoxicity, with the only exception of **G-CDC** which was found to be selectively cytotoxic against HCT116 cancer cells (IC_50_ = 25.3 μM).

In the 2′-deoxyuridine series **dU-UDC** was found cytotoxic towards both K562 and HCT116, with comparable IC_50_ values (21.5 and 23.5 μM, respectively), whereas **dU-*nor*-UDC** showed cytoselectivity towards K562, with IC_50_ = 24.8 μM vs. HCT116 IC_50_ = 43.0 μM (Figure 1, Table 1). No cytotoxic activity was found for the conjugates with TUDCA.

**Figure 2:**
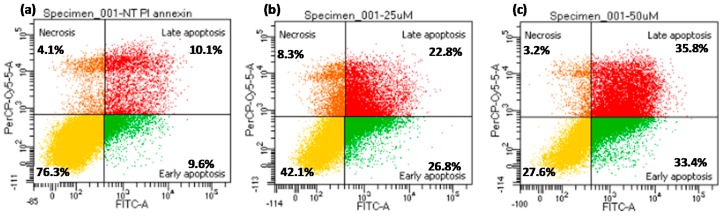
**dA-*nor*-CDC** induced apoptosis in K562 cells after 24 h treatment: (**a**) control: untreated cells; (**b**) **dA-*nor*-CDC** 25 μM; (**c**) **dA-*nor*-CDC** 50 μM.

In this study we also tested in vitro the nucleoside-alkyne derivatives (namely **ALK-A**, **ALK-G**, **ALK-dG** and **ALK-dU**) and the N_3_-BA building blocks (namely **3α-N_3_-CDC**, **3α-N_3_-UDC**, **3α-N_3_-TUDCA**, **23-N_3-_*nor*-CDC** and **23-N_3-_*nor*-UDC**). The nucleoside-alkyne derivatives were found to consistently not be cytotoxic towards any of tested cell lines with the results previously reported for **ALK-dA** [6]. As far as for the **3α-N_3_-**BA is concerned, we found that **3α-N_3_-CDC** and **3α-N_3_-UDC** are active against both cancer cell lines indiscriminately and not active towards the fibroblast cells up to 100 μM, whereas **3α-N_3_-TUDCA** was found to not be cytotoxic against any of the cell lines at the concentrations tested. On the other hand, the **23-N_3_-*nor*-**BA series showed cytotoxicity against the cancer cell lines, with IC_50_ values ≤ 25 μM, and, to a minor extent, also towards the fibroblasts. (Table 1). To determine whether the antiproliferative activity induced by **dA-*nor*-CDC** was related to apoptosis, as previously reported for the corresponding **dA-CDC** conjugate [6], K562 cells were treated with compound **dA-*nor*-CDC** (25 and 50 μM) for 24 h, then assayed by flow cytometry analysis with Annexin V-FITC staining. The results of the cell apoptosis assay indicated that compound **dA-*nor*-CDC** induced apoptosis in a dose dependent manner (Figure 2).

## 3. Discussion

The reported in vitro screening highlighted some compounds with an interesting anticancer activity, with IC_50_ values ≤ 25 μM, which are **dA-*nor*-CDC**, **A-*nor*-CDC**, **dU-UDC** and **dU-*nor*-UDC** with respect to K562 leukemia cells and **dA-*nor*-CDC**, **A-CDC**, **G-CDC** and **dU-UDC** with respect to HCT116 colon carcinoma (Table 1, Figure 1). Among them, only **dA-*nor*-CDC** and **dU-UDC** showed good anti-proliferative activity against both K562 and HCT116, with comparable IC_50_ values. It is worth noting that these two compounds showed also a higher IC_50_ value (around 100 μM) respect to the other conjugates towards the fibroblast cells therefore the lack of cytoselectivity among the selected cancer lines could be related to the greater activity of the compounds (Table 1).

In agreement with our previous data [6] the A/dA-based bioconjugates were confirmed to be potential active anticancer compounds. Besides, the present screening also evidenced G- and U-based conjugates with interesting cytotoxicity/cytoselectivity. Moreover, it can be observed that CDC/*nor*-CDC scaffolds conjugated with A/dA nucleosides showed in all cases a fair cytotoxic activity and cytoselectivity. Conversely, UDC/*nor*-UDC scaffolds showed cytotoxic activity only when coupled with 2′-deoxyuridine (Table 1).

In our previous paper on 2′-deoxyadenosine-BA conjugates, including **dA-CDC** and **dA-UDC** those IC_50_ values are also reported in Table 1 for comparison, we demonstrated that the conjugation of dA with CDC and UDC actually plays a crucial role in the cytotoxic/cytoselective process [6]. Starting from this point we would like to discuss the possible structure-activity relationship in the light of the biological evaluation of the nucleoside-BA conjugates incorporating a triazole moiety herein reported. A marked cytoselectivity trend among the selected cancer lines can be identified in the adenosine series. In fact, compound **A-CDC** is preferentially cytotoxic against HCT116 cells whereas the corresponding **A-*nor*-CDC** derivative showed cytoselectivity towards K562 cells. Therefore, CDC bile acid seems to address the cytoselectivity to HCT116 whereas *nor*-CDC bile acid does the same for K562 cells. This seems to be supported by the data of both the G and dU series where **G-CDC** was found highly cytoselective against the HCT116 and **dU-*nor*-CDC** cytoselective against the K562 as expected in the light of the previous consideration. However, this hypothesis is in contrast with the cytoselectivity of **dA-CDC** that is selective against the K562 unless it is a CDC derivative. The overall data also indicate that the CDC/*nor*-CDC derivatives are more active than the corresponding UDC/*nor*-UDC except for the conjugates with 2′-deoxyuridine (Figure 2). Therefore, the conjugation with a pyrimidine nucleoside seems to improve the anticancer activity of the UDC/*nor*-UDC series. Looking more deeply through the biological data it can be seen that also the sugar nature, deoxy- or ribo-, seems to influence the cytoselectivity being the ribo form more active against the HCT116 in the adenine series (comparison between **dA-CDC** and **A-CDC**) and also in the corresponding guanine series (**G-CDC**) (Table 1). Finally, in the case of TUDCA-conjugates no cytotoxic activity was found, as for the corresponding **3α-N_3_-TUDCA** building block. The overall data herein debated seems to indicate that the cytoselectivity is mainly driven by the BA and can be fine-tuned by the nucleoside nature, i.e., purine or pyrimidine, deoxy- or ribo-.

## 4. Materials and Methods

### 4.1. General Information

Reactions were monitored by TLC on pre-coated silica gel plates (thickness 0.25 mm, Merck, Darmstadt, Germany), and phosphomolybdic acid solution was used as the spray reagent to visualize the steroids. Flash column chromatography was performed on silica gel 60 (230–400 mesh). HPLC-MS analyses were performed on an Agilent 1100 HPLC system (Agilent Tech. Inc., Santa Clara, CA, USA) and an Esquire 3000 Plus mass spectrometer (Bruker, Billerica, MA, USA) using a Zorbax C8 column (4.6 mm × 150 mm, 5 μm) (linear gradient water/CH_3_CN at a 0.5 mL/min flow rate, detection at λ 260 nm). ESI-HRMS were acquired on an Agilent Dual ESI Q TOF 6520 (Agilent Tech. Inc., Santa Clara, CA, USA), in positive-ion mode, using methanol. NMR spectra were recorded for DMSO-*d*_6_ solutions, unless otherwise specified, with a Mercury Plus 400 MHz instrument (Varian, Palo Alto, CA, USA). IR spectra were recorded on a Spectrum 100 FT-IR spectrometer (Perkin–Elmer, Waltham, MA, USA). 8-Br-Adenosine, 8-Br-2′-deoxyadenosine, 8-Br-2′-deoxyguanosine, 8-Br-guanosine, 5-I-2′-deoxyuridine, chenodeoxycholic, ursodeoxycholic, are commercially available compounds that were used without further purification. The corresponding azides, methyl 3α-azido-7α-hydroxy-5β-cholan-24-oate (**3α-N_3_-CDC**), and methyl 3α-azido-7β-hydroxy-5β-cholan-24-oate (**3α-N_3_-UDC**), were prepared according to the literature procedures [6,17].

### 4.2. General Procedure for the Synthesis of Alkynes

Alkynes were prepared following the procedure reported in the literature [16]. In all cases they were obtained in 70–80% yield and no chromatographic purification was necessary. An analytical sample for the characterization analyses was obtained after flash chromatography using CH_2_Cl_2_:MeOH = 9:1 as eluent in all cases.

*8-(1,7-Octadynyl)-adenosine* (**ALK-A**) ^1^H-NMR δ = 8.18 (1H, s, H2), 7.56 (2H, br s; disappeared upon shaking with D_2_O; NH_2_), 5.90 (1H, d, *J* = 7.2 Hz; collapsing to s upon irradiation at δ 4.98; H1′), 5.54 (1H, m; disappeared upon shaking with D_2_O; C5′-OH), 5.39 (1H, d, *J* = 6.4 Hz; collapsing to s upon irradiation at δ 4.98; disappeared upon shaking with D_2_O; C2′-OH), 5.16 (1H, d, *J* = 3.6 Hz; collapsing to s upon irradiation at δ 4.17; disappeared upon shaking with D_2_O; C3′-OH), 4.98 (1H, m; collapsing to dd, *J*_1_ = 7.2 Hz and *J*_2_ = 6.4 Hz, upon irradiation at δ 4.17; H2′), 4.18 (1H, m; H3′), 3.96 (1H, m; H4′), 3.64 (1H, m; H5′), 3.50 (1H, m; H5′′), 2.53 (3H, m), 2.37 (2H, m), 1.64 (4H, m). ^13^C-NMR: δ = 153.7 (CH), 153.4 (q), 149.5 (q), 132.0 (q), 94.7 (q), 94.6 (q), 89.9 (CH), 87.2 (CH), 72.1 (CH), 71.6 (CH), 66.2 (q), 62.8 (CH), 55.5 (CH_2_), 27.5 (CH_2_), 27.3 (CH_2_), 18.7 (CH_2_), 18.5 (CH_2_). MS (ESI, ES+) *m*/*z*: 394 (M + 23), 372 (M + 1).

*8-(1,7-Octadynyl)-guanosine* (**ALK-G**) ^1^H-NMR: δ = 5.75 (1H, d, *J* = 7.2 Hz; collapsing to s upon irradiation at δ 4.90; H1′), 4.98 (1H, m; collapsing to dd, *J*_1_ = 7.2 Hz and *J*_2_ = 6.8 Hz, upon irradiation at δ 4.08; H2′), 4.08–4.05 (1H, m; H3′), 3.94–3.91 (1H, m; H4′), 3.64–3.40 (2H, m; H5′, H5′′), 2.75 (1H, t, *J* = 2.8 Hz), 2.58–2.50 (2H, m), 2.23–2.18 (2H, m), 1.65–1.53 (4H, m). ^13^C-NMR: δ = 163.4 (q), 137.1 (q), 134.6 (q), 131.4 (q), 118.9 (q), 94.5 (q), 89.5 (q), 87.1 (CH), 84.9 (CH), 72.4 (q), 72.1 (CH), 72.1 (CH), 71.8 (CH), 63.3 (CH_2_), 27.8 (CH_2_), 26.7 (CH_2_), 18.8 (CH_2_), 17.9 (CH_2_). MS (ESI, ES+) *m*/*z*: 410 (M + 23), 388 (M + 1).

*8-(1,7-Octadynyl)-2′-deoxyguanosine* (**ALK-dG**) ^1^H-NMR: δ = 6.23–6.18 (1H, m; H1′), 4.40–4.38 (1H, m; H3′), 3.90–3.83 (1H, m; H4′), 3.68–3.60 (1H, m; H5′), 3.58–3.42 (1H, m; H5′′), 3.02–2.95 (1H, m; H2′′), 2.75 (1H, t, *J* = 2.8 Hz), 2.58–2.50 (2H, m), 2.25–2.19 (2H, m), 2.18–1.98 (1H, m; H2′), 1.70–1.52 (4H, m). ^13^C-NMR: δ = 163.4 (q), 137.1 (q), 134.6 (q), 131.4 (q), 118.9 (q), 94.5 (q), 89.5 (q), 87.1 (CH), 84.9 (CH), 72.4 (q), 72.1 (CH), 71.8 (CH), 63.3 (CH_2_), 40.2 (CH_2_), 27.8 (CH_2_), 26.7 (CH_2_), 18.8 (CH_2_), 17.9 (CH_2_). MS (ESI, ES+) *m*/*z*: 394 (M + 23); (ESI, ES−) *m*/*z*: 370 (M − 1).

*5-(1,7-Octadynyl)-2′-deoxyuridine* (**ALK-dU**) ^1^H-NMR: δ = 8.10 (1H, s, H6), 6.08 (1H, dd, *J*_1_ = 6.4 Hz; collapsing to s upon irradiation at δ 2.08; H1′), 5.22 (1H, m; disappeared upon shaking with D_2_O; C5′-OH), 5.07 (1H, m, disappeared upon shaking with D_2_O; C3′-OH), 4.21–4.18 (1H, m; H3′), 3.78 (1H, m; collapsing to d, *J* = 2.8 Hz upon irradiation at δ 3.55; H4′), 3.58–3.53 (2H, m; H5′and H5′′), 2.74 (1H, t, *J* = 2.4 Hz; collapsing to s upon irradiation at δ 2.18), 2.362.32 (2H, m), 2.20–2.17 (2H, m), 2.11–2.06 (2H, m, H2′ and H2′′), 1.60–1.53 (4H, m). ^13^C-NMR: δ = 163.2 (q), 150.1 (q), 143.4 (CH), 99.7 (q), 93.7 (q), 88.1 (CH), 85.3 (CH), 85.1 (q), 72.0 (q), 70.8 (CH), 61.5 (CH_2_), 46.4 (CH_2_), 27.9 (CH_2_), 27.7 (CH_2_), 19.0 (CH_2_), 17.9 (CH_2_). MS (ESI, ES+) *m*/*z*: 665 (2M + 1), 687 (2M + 23), 355 (M + 23), 333 (M + 1).

### 4.3. Synthesis of Diformyloxy-5β-23-iodio-24-norcholanes

3α,7β-Diformyloxy-5β-23-iodo-24-norcholane and 3α,7α-diformyloxy-5β-23-iodo-24*-nor-*cholane were prepared from the corresponding bile acid according to the literature procedure [18]. A mixture of bile acid (2.5 mmol) and formic acid (4 mL) was stirred at 55 °C for 24 h and concentrated in vacuo. The residue was crystallized by adding water to warm EtOH solution and used in the next step. A solution of diformyloxy bile acid (0.5 mmol) and DIH (228 mg, 0.6 mmol) in DCE (4 mL) was irradiated for 2 h under reflux conditions. After chromatography on silica gel (eluent, 0:100 to 50:50 EtOAc/hexane) 23-I-*nor*-UDC and 23-I-*nor*-CDC were obtained.

*3α,7β-Diformyloxy-5β-23-iodio-24-norcholane* (**23-I*-nor-*UDC**). Yield 85% ^1^H-NMR (CDCl_3_): δ = 7.99 (s, 1H), 7.97 (s, 1H), 4.94–4.75 (m, 2H), 3.33–3.23 (m, 1H), 3.12–3.02 (m, 1H), 2.05–1.12 (m, 24H), 0.98 (s, 3H), 0.91 (d, *J* = 6.15 Hz, 3H), 0.64 (s, 3H); ^13^C-NMR (CDCl_3_): δ = 161.0 (q), 160.6 (q), 73.5 (CH), 73.3 (CH), 55.2 (CH), 54.8 (CH), 43.7 (q), 42.0 (CH), 40.1 (CH_2_), 39.8 (CH_2_), 39.7 (CH), 39.4 (CH), 36.9 (CH), 34.3 (CH_2_), 33.9 (q), 32.8 (CH_2_), 32.7 (CH_2_), 28.3 (CH_2_), 26.3 (CH_2_), 25.8 (CH_2_), 23.2 (CH_3_), 21.2 (CH_2_), 17.86 (CH_3_), 12.0 (CH_3_), 5.3 (CH_2_). MS (ESI, ES+) *m*/*z*: 553 (M + 23).

*3α,7α-Diformyloxy-5β-23-iodio-24-norcholane* (**23-I*-nor-*CDC**). Yield 90% ^1^H-NMR (CDCl_3_): δ = 8.08 (s, 1H), 8.02 (s, 1H), 5.03 (br s, 1H), 4.78–4.65 (m, 1H), 3.38–3.23 (m, 1H), 3.17–3.04 (m, 1H), 2.18–1.03 (m, 24H), 0.98 (s, 3H), 0.96 (d, *J* = 6.2 Hz, 3H), 0.64 (s, 3H); ^13^C-NMR (CDCl_3_): δ = 160.8 (q), 74.1 (CH), 71.4 (CH), 55.6 (CH), 50.1 (CH), 42.8 (q), 40.9 (CH), 40.2 (CH_2_), 39.4 (CH_2_), 37.9 (CH), 37.1 (CH), 34.8 (CH_2_), 34.6 (CH), 34.0 (q), 33.8 (CH_2_), 31.5 (CH_2_), 28.0 (CH_2_), 26.8 (CH_2_), 23.5 (CH_2_), 22.7 (CH_3_), 20.6 (CH_2_), 17.9 (CH_3_), 11.8 (CH_3_), 5.2 (CH_2_). MS (ESI, ES+) *m*/*z*: 553 (M + 23).

### 4.4. General Procedure for the Synthesis of Nor-Azides

The 23-iodo derivative (0.5 mmol) was dissolved in DMF (3 mL) and NaN_3_ (4 mmol) was added. The reaction mixture was stirred at room temperature overnight and then poured into water (8 mL) and extracted twice with Et_2_O (12 mL). The combined organic layers were dried over anhydrous Na_2_SO_4_, filtered and concentrated in vacuo to give the diformyloxy azido-compound. The pale yellow solid was treated with 25% NaOH in MeOH at room temperature monitoring by TLC (AcOEt/cyclohexane 1:1) until disappearing of the starting material (2 h for UDC, 12 h for CDC). The corresponding dihydroxy azido derivatives **23-N_3_-*nor*-UDC** and **23-N_3_*-nor*-CDC** were precipitated by adding water to the solution.

*3α,7β-Dihydroxy-5β-23-azido-24-norcholane* (**23-N_3_*-nor-*UDC**). Amorphous white solid, yield 75%; IR: *ν* (cm^−1^) 3593 (O-H), 3447 (O-H), 2970–2855 (C-H), 2086 (N_3_); ^1^H-NMR: δ = 4.43 (d, *J* = 4.5 Hz, 1H), 3.86 (d, *J* = 6.8 Hz, 1H), 3.42–3.31 (m, 1H), 3.29–3.18 (m, 3H), 1.98–1.58 (m, 6H), 1.51–0.91 (m, 18H), 0.89 (d, *J* = 6.4 Hz, 3H), 0.82 (s, 3H), 0.59 (s, 3H); ^13^C-NMR: δ = 69.7 (CH), 69.4 (CH), 55.8 (CH), 54.8 (CH), 48.3 (CH_2_), 43.1 (q), 42.9 (CH), 42.1 (CH), 39.7 (CH_2_), 38.7 (CH), 37.7 (CH_2_), 37.2 (CH_2_), 34.8 (CH_2_), 34.3 (CH_2_), 33.7 (q), 33.1 (CH), 30.2 (CH_2_), 28.2 (CH_2_), 26.7 (CH_2_), 23.3 (CH_3_), 20.8 (CH_2_), 18.4 (CH_3_), 11.9 (CH_3_). MS (ESI, ES+) *m*/*z*: 1191 (3M + 23).

3α,7α-*Dihydroxy-5β-23-azido-24-norcholane* (**23-N_3_*-nor-*CDC**). Amorphous white solid, yield 78%; IR: *ν* (cm^−1^) 3439 (O-H), 2970–2864 (C-H), 2091 (N_3_); ^1^H-NMR: δ = 4.29 (d, *J* = 4.6 Hz, 1H), 4.11 (d, *J* = 3.2 Hz, 1H), 3.61 (br s, 1H), 3.44–3.38 (m, 1H), 3.29–3.08 (m, 2H), 2.22–2.09 (m, 1H), 1.95–1.58 (m, 8H), 1.48–0.96 (m, 15H), 0.89 (d, *J* = 6.5 Hz, 3H), 0.82 (s, 3H), 0.60 (s, 3H); ^13^C-NMR: δ = 70.3 (CH), 66.1 (CH), 55.7 (CH), 50.0 (CH), 48.3 (CH_2_), 42.0 (q), 41.4 (CH), 39.6 (CH_2_), 39.3 (CH_2_), 39.1 (CH), 35.3 (CH_2_), 34.8 (CH_2_), 34.7 (q), 34.2 (CH_2_), 33.2 (CH), 32.3 (CH), 30.5 (CH_2_), 27.9 (CH_2_), 23.1 (CH_2_), 22.7 (CH_3_), 20.2 (CH_2_), 18.2 (CH_3_), 11.6 (CH_3_). MS (ESI, ES+) *m*/*z*: 1191 (3M + 23).

### 4.5. Synthesis of (3α-Azido-7β-hydroxy-5β-cholanoate) *(**3α-N_3_-UDCA**)*

LiOH (1.5 M, 15 mL, 23 mmol) was added to a solution of **3α-N_3_-UDC** (1.0 g, 2.3 mmol) in methanol (10 mL). The mixture was stirred at room temperature for 21 h. Then 2N HCl was added until pH = 4–5 and the solution was extracted with ethyl acetate (2 × 20 mL). The combined organic phase was washed with water, dried over MgSO_4_, and concentrated in vacuo to afford a white powder. Yield 94% ^1^H-NMR: δ = 11.93 (br s, 1H), 3.91 (br s, 1H), 3.41–3.22 (m, 2H), 2.25–0.82 (m, 32H), 0.60 (s, 3H); ^13^C-NMR: δ = 174.8 (q), 69.1 (CH), 60.0 (CH), 55.4 (CH), 54.5 (CH), 42.9 (CH), 42.8 (q), 42.0 (CH), 39.4 (CH_2_), 38.4 (CH), 37.2 (CH_2_), 34.7 (CH), 34.5 (CH_2_), 33.6 (q), 32.7 (CH_2_), 30.6 (CH_2_), 28.0 (CH_2_), 26.6 (CH_2_), 26.0 (CH_2_), 23.1 (CH_3_), 20.7 (CH_2_), 18.2 (CH_3_), 11.9 (CH_3_). MS (ESI, ES+) *m*/*z*: 440 (M + 23).

### 4.6. Synthesis of (3α-Azido-7β-hydroxy-5β-cholan-24-oyl)-2-aminoethanesulfonic Acid *(**3α-N_3_-TUDCA**)*

To a solution of 3α-azido-7β-hydroxy-5β-cholanoate (500 mg, 1.19 mmol) in anhydrous THF (5 mL) stirred at 0 °C were added triethylamine (0.18 mL, 1.3 mmol) and ethyl chloroformate (0.13 mL, 1.3 mmol). After 2 h at room temperature a solution of taurine (136 mg, 1.3 mmol) in NaOH/H_2_O (1 mL, 1.43 mmol) was added. The reaction mixture was stirred at room temperature overnight and then acidified with 5% HCl to pH 1. After evaporation of THF, the mixture was diluted with water and washed with EtOAc. The aqueous phase was extracted with *n*-butanol and the organic layer dried over anhydrous Na_2_SO_4_, filtered and concentrated under reduced pressure to gove the title compound as an amorphous white solid, yield 80%; IR: *ν* (cm^−1^) 3309 (O-H), 2931–2866 (C-H), 2090 (N_3_), 1648 (C=O); ^1^H-NMR: δ = 7.72 (br s, 1H), 6.83–6.80 (m, 1H), 3.98–3.88 (m, 2H), 3.28–3.17 (m, 2H), 3.15–3.01 (m, 2H), 2.68–2.75 (m, 1H), 2.57–2.48 (m, 2H), 2.08–0.93(m, 23H), 0.87 (s, 3H), 0.85 (d, *J* = 6.4 Hz, 3H), 0.59 (s, 3H); ^13^C-NMR: δ = 171.9 (q), 69.0 (CH), 60.0 (q), 55.4 (CH), 54.5 (CH), 50.5 (CH_2_), 45.6 (CH_2_), 42.9 (CH), 42.8 (q), 42.1 (CH), 40.0 (CH), 38.4 (CH), 37.2 (CH_2_), 35.4 (CH_2_), 34.8 (CH), 34.5 (CH_2_), 33.6 (CH_2_), 32.5 (CH_2_), 31.4 (CH_2_), 28.0 (CH_2_), 26.6 (CH_2_), 26.0 (CH_2_), 23.1 (CH_3_), 20.7 (CH_2_), 18.4 (CH_3_), 11.9 (CH_3_). MS (ESI, ES+) *m*/*z*: 547 (M + 23).

### 4.7. General Procedure for the “Click” Reaction

To a solution of the appropriate alkyne **ALK-dA**, **ALK-A**, **ALK-G**, **ALK-dG**, **ALK-dU** (0.03 mmol) in 1.4 mL of a 1:1:1.5 mixture of H_2_O/*tert*-BuOH /THF (*v*/*v*), sodium ascorbate (0.06 mmol) and copper(II) sulfate (0.012 mmol) were added. Then the appropriate azide **3α-N_3_-CDC**, **3α-N_3_-UDC**, **23-N_3_-*nor*-CDC**, **23-N_3_-*nor*-UDC**, **3α-N_3_-TUDCA** (0.045 mmol) was added and the resulting solution was stirred at room temperature overnight.

#### 4.7.1. Method A (Purification of Conjugates with 3α-Azides)

The mixture was concentrated under reduced pressure, added with water and extracted with dichloromethane. The organic layers was dried over Na_2_SO_4_, filtered and concentrated in vacuo. The resulting crude solid was washed three times with Et_2_O.

#### 4.7.2. Method B (Purification of Conjugates with nor-azides)

The mixture was concentrated in vacuo until the complete elimination of THF and *tert*-BuOH. The crude precipitated solid was filtered, washed with water, EtOH, EtOAc and finally dried with Et_2_O.

#### 4.7.3. Method C (Purification of Conjugates with TUDCA-Azides)

The mixture was concentrated under reduced pressure, added with water and extracted with *n*-butanol. The organic layers was dried over Na_2_SO_4_, filtered and concentrated in vacuo. The crude white solid was washed twice with EtOH (10 mL) and dried with Et_2_O.

***A-CDC.*** Colourless syrup, yield 80%; IR: *ν* (cm^−1^) 3418–3315 (O-H), 2928–2866 (C-H), 2241 (C≡C), 1693 (C=O), 1665–1524 (C=C,C=N); ^1^H-NMR: δ = 8.18 (br s, 1H), 7.88 (s, 1H), 7.58 (br s, 2H), 5.98 (d, *J* = 6.83 Hz 1H), 5.61–5.58 (m, 1H), 5.42 (d, *J* = 6.25 Hz 1H), 5.20 (d, *J* = 4.30 Hz, 1H), 5.12–4.98 (m, 1H), 4.32–4.16 (m, 3H), 3.98 (m, 1H), 3.72–3.42 (s, 7H), 2.74–2.59 (m, 5H), 2.38–2.16 (m, 3H), 2.01–0.95 (m, 25H), 0.90 (s, 3H), 0.86 (d, *J* = 6.44 Hz, 3H), 0.60 (s, 3H); ^13^C-NMR: δ = 173.8 (q), 156.0 (q), 153.2 (CH), 148.3 (q), 146.1 (q), 134.0 (q), 120.0 (q), 119.9 (CH), 97.4 (q), 89.3 (CH), 86.6 (CH), 71.5 (CH), 71.08 (CH), 70.3 (q), 66.1 (CH), 62.2 (CH_2_), 60.1 (CH), 55.4 (CH), 51.2 (CH_3_), 49.9 (CH), 41.9 (CH), 41.7 (q), 40.1 (CH_2_), 37.7 (CH_2_), 35.4 (CH_2_), 34.9 (q), 34.8 (CH), 34.4 (CH_2_), 32.2 (CH), 30.6 (CH_2_), 30.4 (CH_2_), 28.2 (CH_2_), 27.7 (CH_2_), 27.2 (CH_2_), 27.1 (CH_2_), 24.6 (CH_2_), 23.1 (CH_2_), 22.6 (CH_3_), 20.3 (CH_2_), 18.4 (CH_2_), 18.1 (CH_3_), 11.6 (CH_3_). HRMS calculated for [C_43_H_62_N_8_O_7_ + H]^+^ 803.4814, found 803.4819.

***A-UDC.*** Colourless syrup, yield 78%; IR: *ν* (cm^−1^) 3411 (O-H), 2926–2865 (C–H), 2240 (C≡C), 1693 (C=O), 1660–1524 (C=C,C=N); ^1^H-NMR: δ = 8.18 (br s, 1H), 8.02 (s, 1H), 7.58 (br s, 2H), 5.98 (d, *J* = 6.83 Hz, 1H), 5.61–5.58 (m, 1H), 5.42 (d, *J* = 6.23 Hz, 1H), 5.20 (d, *J* = 4.31 Hz, 1H), 5.12–4.98 (m, 1H), 4.38 (br, s, 1H), 4.18 (br, s, 1H), 3.98 (br, s, 1H), 3.91 (br, s, 1H), 3.71–3.62 (m, 1H), 3.58–3.45 (m, 4H), 2.74–2.59 (m, 5H), 2.38–2.11 (m, 5H), 2.01–0.95 (m, 25H), 0.92 (s, 3H), 0.84 (d, *J* = 6.44 Hz, 3H), 0.60 (s, 3H); ^13^C-NMR: δ = 174.2 (q), 156.4 (q), 153.4 (CH), 148.7 (q), 142.8 (q), 133.9 (q), 120.4 (q), 120.2 (CH), 98.0 (q), 88.8 (CH), 85.6 (CH), 71.8 (q), 70.8 (CH), 66.5 (CH), 62.7 (CH_2_), 60.5 (CH), 55.9 (CH), 51.6 (CH_3_), 50.4 (CH), 42.4 (q), 42.1 (CH), 39.6 (CH), 38.0 (CH_2_), 37.7 (CH_2_), 35.9 (CH_2_), 35.3 (CH), 35.3 (CH_2_), 34.9 (q), 32.7 (CH_2_), 31.1 (CH), 30.8 (CH_2_), 28.7 (CH_2_), 28.2 (CH_2_), 27.6 (CH_2_), 27.5 (CH_2_), 25.0 (CH_2_), 23.5 (CH_2_), 23.1 (CH_2_), 20.7 (CH_3_), 18.8 (CH_2_), 18.6 (CH_3_), 12.1 (CH_3_); HRMS calculated for [C_43_H_62_N_8_O_7_ + H]^+^ 803.4814, found 803.4829.

***dG-CDC.*** Colourless syrup, yield 76%; IR: *ν* (cm^−1^) 3327 (O-H), 2933–2865 (C-H), 2243 (C≡C), 1692 (C=O), 1629–1568 (C=C,C=N); ^1^H-NMR: δ = 10.82 (br, s, 1H), 7.85 (s, 1H), 6.57 (br, s, 2H), 6.25–6.19 (m, 1H), 5.22 (br, s, 1H), 4.86 (br, s, 1H), 4.4–4.16 (m, 3H), 3.8 (m, 1H), 3.6–3.58 (m, 2H), 3.59 (s, 3H), 3.57–3.42 (m, 2H), 3.15–2.99 (m, 1H), 2.71–2.62 (m, 2H), 2.53 (m, 2H), 2.38–2.01 (m, 5H), 1.92–0.99 (m, 25H), 0.89 (s, 3H), 0.86 (d, *J* = 6.40 Hz, 3H), 0.60 (s, 3H); ^13^C-NMR: δ = 173.6 (q), 156.5 (q), 153.6 (q), 150.5 (q), 146.3 (q), 130.1 (q), 121.6 (CH), 116.8 (q), 95.2 (q), 87.6 (CH), 84.6 (CH), 70.9 (q), 70.6 (CH), 70.5 (CH), 66.0 (CH), 62.0 (CH_2_), 60.0 (CH), 55.4 (CH), 51.1 (CH_3_), 49.8 (CH), 41.9 (CH), 41.6 (q), 40.0 (CH_2_), 39.6 (CH), 38.8 (CH_2_), 37.1 (CH_2_), 35.4 (CH_2_), 34.7 (q), 34.3 (CH_2_), 32.1 (CH), 30.6 (CH_2_), 30.3 (CH_2_), 28.1 (CH_2_), 27.7 (CH_2_), 27.1 (CH_2_), 24.5 (CH_2_), 23.0 (CH_2_), 22.6 (CH_3_), 20.2 (CH_2_), 18.3 (CH_2_), 18.2 (CH_3_), 11.6 (CH_3_). HRMS calculated for [C_43_H_62_N_8_O_7_ + H]^+^ 803.4814, found 803.4815.

***dG-UDC.*** Colourless syrup, yield 78%; IR: *ν* (cm^−1^) 3312 (O-H), 2929–2860 (C-H), 2240 (C≡C), 1686 (C=O), 1601–1565 (C=C,C=N); ^1^H-NMR: δ = 10.78 (br, s, 1H), 7.99 (s, 1H), 6.48 (br, s, 2H), 6.28–6.18 (m, 1H), 5.22 (br, s, 1H), 4.93–4.82 (m, 1H), 4,38 (br, s, 2H), 3.91 (d, *J* = 6.45 Hz, 1H), 3.79–3.75 (m, 1H), 3.62–3.41 (m, 5H), 3.19–2.98 (m, 1H), 2.68–0.99 (m, 36H), 0.97 (s, 3H), 0.86 (d, *J* = 6.44 Hz, 3H), 0.59 (s, 3H); ^13^C-NMR: δ = 173.7 (q), 158.3 (q), 155.9 (q), 153.6 (q), 150.5 (q), 149.9 (q), 129.5 (q), 119.8 (CH), 94.0 (q), 87.6 (CH), 83.6 (CH), 71.8 (q), 71.0 (CH), 68.9 (CH), 68.0 (CH), 62.0 (CH_2_), 59.5 (CH), 55.2 (CH), 54.5 (CH), 51.1 (CH_3_), 42.9 (CH), 42.4 (CH), 41.8 (q), 40.0 (CH_2_), 37.2 (CH_2_), 36.8 (CH_2_), 34.9 (CH_2_), 34.7 (CH), 34.0 (q), 33.7 (CH_2_), 30.6 (CH_2_), 30.2 (CH_2_), 28.1 (CH_2_), 27.5 (CH_2_), 27.1 (CH_2_), 26.5 (CH_2_), 24.5 (CH_2_), 23.8 (CH_2_), 23.1 (CH_3_), 20.8 (CH_2_), 18.2 (CH_2_), 18.1 (CH_3_), 11.9 (CH_3_). HRMS calculated for [C_43_H_62_N_8_O_7_ + H]^+^ 803.4814, found 803.4816.

***G-CDC.*** Colourless syrup, yield 80%; IR: *ν* (cm^−1^) 3330 (O-H), 2918–2850 (C-H), 2238 (C≡C), 1736 (C=O), 1645–1580 (C=C,C=N); ^1^H-NMR: δ = 10.78 (br, s, 1H), 7.88 (s, 1H), 6.48 (br, s, 2H), 5.78 (d, *J* = 6.44 Hz, 1H), 5.40 (d, *J* = 6.25 Hz, 1H), 5.11 (d, *J* = 4.88, 1H), 4.99–4.82 (m, 2H), 4,32–4.18 (m, 2H), 4.15 (br, s, 1H), 3.82 (br, s, 1H), 3.63–3.41 (m, 4H), 2.75–2.15 (m, 5H), 1.96–0.99 (m, 31 H), 0.88 (s, 3H), 0.84 (d, *J* = 6.44 Hz, 3H), 0.60 (s, 3H); ^13^C-NMR: δ = 173.7 (q), 157.4 (q), 155.9 (q), 153.7 (q), 150.7 (q), 130.2 (q), 119.9 (CH), 116.8 (q), 95.2 (q), 88.3 (CH), 85.5 (CH), 70.9 (q), 70.6 (CH), 70.5 (CH), 66.0 (CH), 62.0 (CH_2_), 60.0 (CH), 55.4 (CH), 51.1 (CH_3_), 49.8 (CH), 41.9 (CH), 41.6 (q), 40.0 (CH_2_), 39.6 (CH), 38.8 (CH_2_), 37.1 (CH_2_), 35.4 (CH_2_), 34.8 (CH), 34.7 (q), 34.3 (CH_2_), 32.1 (CH), 30.6 (CH_2_), 30.3 (CH_2_), 28.1 (CH_2_), 27.7 (CH_2_), 27.1 (CH_2_), 24.5 (CH_2_), 23.0 (CH_2_), 22.5 (CH_3_), 20.2 (CH_2_), 18.3 (CH_2_), 18.1 (CH_3_), 11.6 (CH_3_). HRMS calculated for [C_43_H_62_N_8_O_8_ + H]^+^ 819.4763, found 819.4768.

***G-UDC.*** Light yellow syrup, yield 78%; IR: *ν* (cm^−1^) 3327 (O-H), 2930–2875 (C-H), 2238 (C≡C), 1735 (C=O), 1645–1572 (C=C,C=N); ^1^H-NMR: δ = 10.78 (br, s, 1H), 8.02 (s, 1H), 6.48 (br, s, 2H), 5.78 (d, *J* = 6.43 Hz, 1H), 5.40 (d, *J* = 6.24 Hz, 1H), 5.11 (d, *J* = 4.88, 1H), 4.99–4.82 (m, 2H), 4.42–4.35(m, 1H), 4,18–4.12 (m, 1H), 3.91 (d, *J* = 6.64 Hz, 1H), 3.823.78 (m, 1H), 3.68–3.38 (m, 6H), 2.71–0.98 (m, 34H), 0.92 (s, 3H), 0.83 (d, *J* = 6.44 Hz, 3H), 0.58 (s, 3H); ^13^C-NMR: δ = 173.5 (q), 156.0 (q), 153.7 (q), 150.7 (q), 146.3 (q), 130.2 (q), 121.6 (CH), 116.8 (q), 95.2 (q), 88.3 (CH), 85.5 (CH), 71.8 (q), 71.0 (CH), 68.9 (CH), 68.0 (CH), 62.0 (CH_2_), 59.5 (CH), 55.2 (CH), 54.5 (CH), 51.1 (CH_3_), 42.9 (CH), 42.4 (CH), 41.8 (q), 40.0 (CH_2_), 37.2 (CH_2_), 36.8 (CH_2_), 34.9 (CH_2_), 34.7 (CH), 34.0 (q), 33.7 (CH_2_), 30.6 (CH_2_), 30.2 (CH_2_), 28.1 (CH_2_), 27.5 (CH_2_), 27.1 (CH_2_), 26.5 (CH_2_), 24.5 (CH_2_), 23.8 (CH_2_), 23.1 (CH_3_), 20.8 (CH_2_), 18.2 (CH_2_), 18.1 (CH_3_), 11.9 (CH_3_). HRMS calculated for [C_43_H_62_N_8_O_7_ + H]^+^ 819.4763, found 819.4766.

***dU-CDC.*** Amorphous white solid, yield 90%; IR: *ν* (cm^−1^) 3440–3310 (O-H), 2930–2861 (C-H), 2244 (C≡C), 1693 (C=O), 1633–1565 (C=C,C=N); ^1^H-NMR: δ = 8.62 (s, 1H), 7.81 (s, 1H), 6.42 (s, 1H), 6.17–6.12 (m, 1H), 5.28 (d, *J* = 4.3 Hz, 1H), 5.14–5.09 (m, 1H), 4.24–4.18 (m, 3H), 3.88 (q, *J* = 3.71 Hz, 1H), 3.67–3.58 (m, 3H), 3.57 (s, 3H), 2.78–2.58 (m, 5H), 2.40–2.24 (m, 2H), 2.23–2.13 (m, 1H), 2.08–1.98 (m, 1H), 1.92–0.95 (m, 27H), 0.89 (s, 3H), 0.86 (d, *J* = 6.44 Hz, 3H), 0.60 (s, 3H); ^13^C-NMR: δ = 173.8 (q), 171.2 (q), 158.1 (q), 153.8 (q), 146.1 (q), 136.8 (CH), 119.8 (CH), 106.4 (q) , 99.9 (q), 88.1 (CH), 87.4 (CH), 69.7 (CH), 66.1 (CH), 60.8 (CH), 60.1 (CH_2_), 55.5 (CH), 51.2 (CH), 49.9 (CH_3_), 42.0 (q), 41.7 (CH), 41.21 (CH), 37.3 (CH_2_), 35.4 (CH_2_), 34.9 (CH), 34.8 (CH_2_), 34.4 (q), 32.2 (CH), 30.7 (CH_2_), 30.4 (CH_2_), 28.3 (CH_2_), 27.8 (CH_2_), 27.2 (CH_2_), 27.1 (CH_2_), 26.0 (CH_2_), 24.7 (CH_2_), 23.1 (CH_2_), 22.6 (CH_3_), 20.3 (CH_2_), 18.2 (CH_3_), 11.7 (CH_3_); HRMS calculated for [C_42_H_61_N_5_O_8_ + H]^+^ 764.4592, found 764.4602.

***dU-UDC.*** Amorphous white solid, yield 70%; IR: *ν* (cm^−1^) 3447–3309 (O-H), 3016–2942 (C-H), 2254 (C≡C), 1690 (C=O), 1645–1522 (C=C,C=N); ^1^H-NMR: δ = 11.58 (s, 1H), 8.18 (s, 1H), 7.98 (s, 1H), 6.12–6.07 (m, 1H), 5.28–5.21 (m, 1H), 5.18–5.15 (m, 1H), 4.42–4.28 (m, 1H), 4.21 (br s, 1H), 3.95 (d, *J* = 6.64 Hz, 1H), 3.78 (br s, 1H), 3.65–3.48 (m, 4H), 2.65–2.58 (m, 2H), 2.41–0.98 (m, 36H), 0.95 (s, 3H), 0.85 (d, *J* = 6.44 Hz, 3H), 0.62 (s, 3H); ^13^C-NMR: δ = 175.4 (q), 171.8 (q), 162.2 (q), 149.9 (q), 146.7 (q), 143.2 (CH), 120.3 (CH), 99.4 (q), 88.0 (CH), 85.0 (CH), 80.7 (CH), 70.6 (CH), 69.5 (CH), 61.4 (CH_2_), 60.0 (CH), 55.8 (CH), 51.6 (CH_3_), 43.5 (CH), 43.0 (CH), 42.5 (q), 40.5 (CH_2_), 40.0 (CH_2_), 38.8 (CH), 37.74 (CH_2_), 35.4 (CH_2_), 35.2 (CH), 34.6 (CH_2_), 34.3 (q), 31.2 (CH_2_), 30.9 (CH_2_), 29.5 (CH_2_), 28.6 (CH_2_), 28.2 (CH_2_), 27.1 (CH_2_), 25.1 (CH_2_), 23.6 (CH_3_), 21.4 (CH_2_), 19.0 (CH_2_), 18.7 (CH_3_), 12.4 (CH_3_); HRMS calculated for [C_42_H_61_N_5_O_8_ + H]^+^ 764.4592, found 764.4600.

***dA-nor-CDC.*** Light yellow syrup, yield 68%; IR: *ν* (cm^−1^) 3440–3332 (O-H), 2926–2865 (C-H), 2242 (C≡C), 1650–1570 (C=C,C=N); ^1^H-NMR: δ = 8.16 (br, s, 1H), 7.89 (s, 1H), 7.58 (br, s, 2H), 6.42–6.38 (m, 1H), 5.43–5.39 (m, 1H), 5.32 (d, *J* = 4.30 Hz, 1H), 4.45 (br, s, 1H), 4.36–4.22 (m, 3H), 4.08 (d, *J* = 4.12 Hz, 1H), 3.90–3.85 (m, 1H), 3.72–3.42 (m, 3H), 3.20–3.03 (m, 2H), 2.71–2.57 (m, 4H), 2.19–2.09 (m, 2H), 1.97–0.99 (m, 27 H), 0.91 (d, *J* = 6.25 Hz, 3H), 0.75 (s, 3H), 0.48 (s, 3H); ^13^C-NMR: δ = 155.8 (q), 152.9 (CH), 148.2 (q), 146.3 (q), 133.3 (q), 121.6 (CH), 119.0 (q), 97.4 (q), 88.2 (CH), 85.1 (CH), 71.3 (CH), 70.2 (CH), 69.2 (q), 66.0 (CH), 62.1 (CH_2_), 55.2 (CH), 50.1 (CH_2_), 49.8 (CH), 46.8 (CH_2_), 41.8 (q), 41.3 (CH), 40.3 (CH), 39.2 (CH_2_), 37.5 (CH_2_), 36.0 (CH_2_), 35.2 (CH_2_), 34.6 (q), 34.5 (CH_2_), 32.9 (CH), 32.1 (CH), 30.4 (CH_2_), 28.0 (CH_2_), 27.7 (CH_2_), 26.7 (CH_2_), 24.2 (CH_2_), 22.9 (CH_2_), 22.5 (CH_3_), 20.0 (CH_2_), 18.1 (CH_2_), 18.1 (CH_3_), 11.3 (CH_3_); HRMS calculated for [C_41_H_60_N_8_O_5_ + H]^+^ 745.4759, found 745.4767.

***dA-nor-UDC.*** Light yellow syrup, 72%; IR: *ν* (cm^−1^) 3440–3317 (O-H), 2928–2861 (C-H), 2243 (C≡C), 1603–1569 (C=C,C=N); ^1^H-NMR: δ = 8.18 (br, s, 1H), 7.88 (s, 1H), 7.55 (br, s, 2H), 6.42–6.39 (m, 1H), 5.42–5.39 (m, 1H), 5.37 (d, *J* = 4.30 Hz, 1H), 4.43 (br, s, 2H), 4.38–4.20 (m, 2H), 3.92–3.81 (m, 2H), 3.70–3.61 (m, 1H), 3.53–3.42 (m, 1H), 3.38–3.18 (m, 3H), 3.17–3.04 (m, 1H), 2.72–2.57 (m, 4H), 2.20–2.11 (m, 1H), 1.97–0.99 (m, 27 H), 0.91 (d, *J* = 6.25 Hz, 3H), 0.81 (s, 3H), 0.52 (s, 3H); ^13^C-NMR: δ = 156.4 (q), 153.3 (CH), 148.7 (q), 147.0 (q), 133.9 (q), 122.3 (CH), 120.0 (q), 97.9 (q), 88.8 (CH), 85.7 (CH), 71.8 (CH), 70.8 (q) 70.2 (CH), 69.8 (CH), 62.7 (CH_2_), 56.2 (CH), 54.9 (CH), 48.8 (CH_2_), 47.5 (CH_2_), 43.5 (q), 43.4 (CH), 42.6 (CH), 40.0 (CH_2_), 39.1 (CH), 38.1 (CH_2_), 37.7 (CH_2_), 36.7 (CH_2_), 35.2 (CH_2_), 34.7 (CH_2_), 34.1 (q), 33.4 (CH), 30.7 (CH_2_), 28.6 (CH_2_), 27.3 (CH_2_), 27.1 (CH_2_), 24.8 (CH_2_), 23.7 (CH_3_), 21.2 (CH_2_), 18.9 (CH_3_), 18.8 (CH_2_), 12.3 (CH_3_); HRMS calculated for [C_41_H_60_N_8_O_5_ + H]^+^ 745.4759, found 745.4765.

***A-nor-CDC.*** Amorphous white solid, yield 68%; IR: *ν* (cm^−1^) 3443–3318 (O-H), 2932–2863 (C-H), 2241 (C≡C), 1640–1570 (C=C,C=N); ^1^H-NMR: δ = 8.12 (s, 1H), 7.87 (s, 1H), 7.58 (br, s, 2H), 5.92 (d, *J* = 6.83 Hz, 1H), 5.60–5.55 (m, 1H), 5.41 (d, *J* = 6.25 Hz, 1H), 5.20 (d, *J* = 4.30 Hz, 1H), 5.00–4.96 (m, 1H), 4.32–4.28 (m, 2H), 4.19–4.12 (m, 1H), 4.08 (d, *J* = 4.12 Hz, 1H), 3.98–3.96 (m, 1H), 3.72–3.44 (m, 3H), 3.20–3.12 (m, 1H), 2.68–2.57 (m, 4H), 2.38–2.16 (m, 3H), 1.98–0.99 (m, 26H), 0.95 (d, *J* = 6.25 Hz, 3H), 0.79 (s, 3H), 0.52 (s, 3H); ^13^C-NMR: δ = 155.8 (q), 152.9 (CH), 148.1 (q), 146.3 (q), 133.9 (q), 121.6 (CH), 119.0 (q), 97.2 (q), 89.2 (CH), 86.5 (CH), 71.4 (CH), 70.9 (CH), 70.2 (CH), 69.5 (q), 66.0 (CH), 62.1 (CH_2_), 55.2 (CH), 49.9 (CH), 46.8 (CH_2_), 41.8 (q), 41.2 (CH), 40.0 (CH), 36.1 (CH_2_), 35.2 (CH_2_), 34.4 (q), 34.6 (CH_2_), 33.0 (CH), 32.1 (CH), 30.4 (CH_2_), 28.9 (CH_2_), 28.6 (CH_2_), 28.0 (CH_2_), 27.7 (CH_2_), 26.8 (CH_2_), 24.3 (CH_2_), 23.0 (CH_2_), 22.6 (CH_3_), 20.1 (CH_2_), 18.2 (CH_2_), 18.2 (CH_3_), 11.4 (CH_3_); HRMS calculated for [C_41_H_60_N_8_O_6_ + H]^+^ 761.4708, found 761.4704.

***A-nor-UDC.*** Amorphous white solid, yield 71%; IR: *ν* (cm^−1^) 3440–3318 (O-H), 2930–2863 (C-H), 2247 (C≡C), 1640–1570 (C=C,C=N); ^1^H-NMR: δ = 8.20 (br, s, 1H), 7.89 (s, 1H), 7.58 (br, s, 2H), 5.92 (d, *J* = 6.83 Hz, 1H), 5.68–5.59 (m, 1H), 5.41 (d, *J* = 6.25 Hz, 1H), 5.22 (d, *J* = 4.30 Hz, 1H), 5.00–4.96 (m, 1H), 4.52 (d, *J* = 4.30 Hz, 1H), 4.35–4.12 (m, 3H), 3.98–3.88 (m, 3H), 3.80–3.44 (m, 3H), 2.71–2.52 (m, 4H), 1.97–0.99 (m, 28H), 0.93 (d, *J* = 6.25 Hz, 3H), 0.82 (s, 3H), 0.53 (s, 3H); ^13^C-NMR: δ = 156.4 (q), 153.5 (CH), 148.7 (q), 146.8 (q), 134.4 (q), 122.3 (CH), 119.5 (q), 97.8 (q), 89.7 (CH), 87.0 (CH), 72.0 (CH), 71.4 (CH), 70.7 (q), 70.1 (CH), 69.9 (CH), 62.6 (CH_2_), 56.2 (CH), 54.9 (CH), 47.4 (CH_2_), 43.5 (q), 43.4 (CH), 42.6 (CH), 39.1 (CH), 38.1 (CH_2_), 37.7 (CH_2_), 36.7 (CH_2_), 35.3 (CH_2_), 34.2 (q), 33.4 (CH), 30.6 (CH_2_), 28.6 (CH_2_), 27.3 (CH_2_), 27.1 (CH_2_), 24.8 (CH_2_), 23.7 (CH_3_), 21.2 (CH_2_), 18.9 (CH_3_), 18.8 (CH_2_), 18.3 (CH_2_), 12.3 (CH_3_); HRMS calculated for [C_41_H_60_N_8_O_6_ + H]^+^ 761.4708, found 761.4715.

***dG-nor-CDC.*** Amorphous white solid, yield 64%; IR: *ν* (cm^−1^) 3402–3320 (O-H), 2924–2850 (C-H), 2233 (C≡C), 1688 (C=O), 1609–1523 (C=C,C=N); ^1^H-NMR: δ = 10.78 (br, s, 1H), 7.88 (s, 1H), 6.49 (br, s, 2H), 6.24–6.18 (m, 1H), 5.23 (br, s, 1H), 4.91 (br, s, 1H), 4.42–4.21 (m, 4H), 4.08 (br, s, 1H), 3.82–3.75 (m, 1H), 3.64–3.41 (m, 3H), 3.11–2.98 (m, 2H), 2.69–2.59 (m, 2H), 2.19–0.98 (m, 31H), 0.98 (d, *J* = 6.25 Hz, 3H), 0.78 (s, 3H), 0.52 (s, 3H); ^13^C-NMR: δ = 156.0 (q), 153.6 (q), 150.4 (q), 146.3 (q), 129.6 (q), 121.6 (CH), 116.8 (q), 95.1 (q), 87.6 (CH), 83.6 (CH), 71.4 (CH), 70.2 (CH), 69.9 (q), 66.0 (CH), 62.0 (CH_2_), 55.2 (CH), 49.8 (CH), 46.8 (CH_2_), 41.8 (q), 41.3 (CH), 40.3 (CH), 39.6 (CH_2_), 39.4 (CH), 38.9 (CH_2_), 36.8 (CH_2_), 36.0 (CH_2_), 35.2 (CH_2_), 34.6 (CH_2_), 32.9 (q), 32.1 (CH), 30.4 (CH_2_), 28.0 (CH_2_), 27.7 (CH_2_), 26.8 (CH_2_), 24.2 (CH_2_), 22.9 (CH_2_), 22.6 (CH_3_), 20.1 (CH_2_), 18.2 (CH_2_), 18.1 (CH_3_), 11.4 (CH_3_). HRMS calculated for [C_41_H_60_N_8_O_6_ + H]^+^ 761.4708, found 761.4705.

***dG-nor-UDC.*** Amorphous white solid, yield 65%; IR: *ν* (cm^−1^) 3405–3322 (O-H), 2932–2852 (C-H), 2243 (C≡C), 1687 (C=O), 1645–1523 (C=C,C=N); ^1^H-NMR: δ = 7.87 (s, 1H), 6.48 (br, s, 2H), 6.25–6.19 (m, 1H), 5.22 (br, s, 1H), 4.89 (br, s, 1H), 4,48–4.22 (m, 4H), 3.90–3.75 (m, 3H), 3.62–3.41 (m, 2H), 3.09–2.98 (m, 1H), 2.75–2.62 (m, 2H), 2.32–0.99 (m, 33H), 0.98 (d, *J* = 6.25 Hz, 3H), 0.88 (s, 3H), 0.57 (s, 3H); ^13^C-NMR: δ = 155.9 (q), 153.5 (q), 150.4 (q), 146.3 (q), 129.7 (q), 121.7 (CH), 116.7 (q), 95.2 (q), 87.6 (CH), 83.6 (CH), 71.0 (CH), 69.9 (q), 69.6 (CH), 69.3 (CH), 62.0 (CH_2_), 55.7 (CH), 54.3 (CH), 46.9 (CH_2_), 42.9 (q), 42.8 (CH), 42.0 (CH), 39.5 (CH_2_), 38.7 (CH), 37.6 (CH_2_), 37.1 (CH_2_), 36.8 (CH_2_), 36.1 (CH_2_), 34.7 (CH_2_), 33.6 (q), 32.8 (CH), 30.1 (CH_2_), 28.9 (CH_2_), 28.0 (CH_2_), 26.8 (CH_2_), 26.5 (CH_2_), 24.2 (CH_2_), 23.2 (CH_3_), 20.7 (CH_2_), 18.3 (CH_3_), 18.2 (CH_2_), 11.8 (CH_3_). HRMS calculated for [C_41_H_60_N_8_O_6_ + H]^+^ 761.4708, found 761.4705.

***G-nor-CDC.*** Light yellow syrup, yield 69%; IR: *ν* (cm^−1^) 3409–3310 (O-H), 2926–2857 (C-H), 2243 (C≡C), 1693 (C=O), 1640–1526 (C=C,C=N); ^1^H-NMR: δ = 10.87 (br, s, 1H), 7.89 (s, 1H), 6.52 (br, s, 2H), 5.78 (d, *J* = 6.44 Hz, 1H), 5.40 (d, *J* = 6.25 Hz, 1H), 5,05 (br, s, 1H), 4.99–4.84 (m, 2H), 4.38–4.21 (m, 4H), 4.09 (br, s, 2H), 3.83 (br, s, 1H), 3.68–3.55 (m, 2H), 3.54–3.43 (m, 1H), 3.21–3.09 (m, 2H), 2.70–2.60 (m, 2H), 2.55–2.48 (m, 2H), 2.22–2.15 (m, 2H), 1.98–1.02 (m, 23H), 0.93 (d, *J* = 6.25 Hz, 3H), 0.79 (s, 3H), 0.52 (s, 3H); ^13^C-NMR: δ = 156.0 (q), 153.6 (q), 150.7 (q), 146.4 (q), 130.3 (q), 121.6 (CH), 116.9 (q), 95.2 (q), 88.4 (CH), 85.5 (CH), 72.0 (CH), 70.2 (CH), 69.8 (q), 66.0 (CH), 62.1 (CH_2_), 55.5 (CH), 49.9 (CH), 48.2 (CH_2_), 41.9 (CH), 41.7 (q), 41.3 (CH), 41.0 (CH), 39.6 (CH_2_), 36.1 (CH), 35.2 (CH_2_), 34.6 (CH_2_), 34.1 (CH_2_), 33.1 (q), 32.1 (CH), 30.4 (CH_2_), 28.0 (CH_2_), 27.8 (CH_2_), 27.0 (CH_2_), 26.5 (CH_2_), 24.3 (CH_2_), 23.0 (CH_2_), 22.6 (CH_3_), 20.1 (CH_2_), 18.1 (CH_3_), 18.0 (CH_2_), 11.5 (CH_3_); HRMS calculated for [C_41_H_60_N_8_O_7_ + H]^+^ 777.4657, found 777.4657.

***G-nor-UDC.*** Light yellow syrup, yield 68%; IR: *ν* (cm^−1^) 3413–3314 (O-H), 2930–2860 (C-H), 2238 (C≡C), 1693 (C=O), 1640–1526 (C=C,C=N); ^1^H-NMR: δ = 10.79 (br, s, 1H), 7.88 (s, 1H), 6.50 (br, s, 2H), 5.78 (d, *J* = 6.44 Hz, 1H), 5.40 (d, *J* = 6.25 Hz, 1H), 5,05 (br, s, 1H), 4.99–4.83 (m, 2H), 4.42 (br, s, 1H), 4.39–4.18 (m, 2H), 4.04 (br, s, 1H), 3.98–3.78 (m, 2H), 3.72–3.58 (m, 1H), 3.57–3.42 (m, 1H), 2.78–2.45 (m, 4H), 1.98–0.99 (m, 30H), 0.98 (d, *J* = 6.25 Hz, 3H), 0.82 (s, 3H), 0.58 (s, 3H); ^13^C-NMR: δ = 156.0 (q), 153.8 (q), 150.7 (q), 146.4 (q), 130.3 (q), 121.8 (CH), 116.9 (q), 95.1 (q), 88.4 (CH), 85.6 (CH), 71.0 (q), 70.7 (CH), 70.6 (CH), 69.7 (CH), 69.4 (CH), 62.1 (CH_2_), 55.8 (CH), 54.5 (CH), 47.0 (CH_2_), 43.1 (q), 43.0 (CH), 42.2 (CH), 40.0 (CH_2_), 38.7 (CH), 37.7 (CH_2_), 37.3 (CH_2_), 36.2 (CH_2_), 34.8 (CH_2_), 33.7 (q), 33.0 (CH), 30.2 (CH_2_), 28.2 (CH_2_), 27.0 (CH_2_), 26.7 (CH_2_), 24.4 (CH_2_), 23.3 (CH_3_), 20.8 (CH_2_), 18.4 (CH_3_), 18.3 (CH_2_), 11.9 (CH_3_); HRMS calculated for [C_41_H_60_N_8_O_7_ + H]^+^ 776.4658, found 776.4668.

***dU-nor-CDC.*** Light yellow syrup, yield 71%; IR: *ν* (cm^−1^) 3446 (O-H), 3100–2850 (C-H), 2255 (C≡C), 1665 (C=O), 1634–1565 (C=C,C=N); ^1^H-NMR: δ = 8.65 (s, 1H), 7.82 (s, 1H), 6.39 (s, 1H), 6.19–6.09 (m, 1H), 5.23 (br, s, 1H), 5.10 (br, s, 1H), 4.38–4.17 (m, 3H), 4.05 (br, s, 1H), 3.86 (br, s, 1H), 3.69–3.52 (m, 3H), 3.21–3.04 (m, 1H), 2.71–2.58 (m, 3H), 2.39–2.25 (m, 1H), 2.21–2.03 (m, 1H), 2.03–0.99 (m, 30 H), 0.93 (d, *J* = 6.25 Hz, 3H), 0.79 (s, 3H), 0.44 (s, 3H); ^13^C-NMR: δ = 158.0 (q), 153.6 (q), 146.3 (q), 136.4 (CH), 121.6 (CH), 106.2 (q), 99.7 (q), 88.0 (CH), 87.3 (CH), 70.2 (CH), 69.6 (CH), 69.5 (q), 66.0 (CH), 60.7 (CH_2_), 55.2 (CH), 49.9 (CH), 46.8 (CH_2_), 41.8 (q), 41.3 (CH), 41.1 (CH_2_), 40.3 (CH), 39.2 (CH_2_), 39.0 (CH_2_), 36.0 (CH_2_), 35.2 (CH_2_), 34.7 (q), 34.6 (CH_2_), 32.9 (CH), 32.1 (CH), 30.4 (CH_2_), 28.1 (CH_2_), 27.7 (CH_2_), 27.0 (CH_2_), 25.6 (CH_2_), 24.4 (CH_2_), 22.9 (CH_2_), 22.6 (CH_3_), 20.1 (CH_2_), 18.1 (CH_3_), 11.3 (CH_3_); HRMS calculated for [C_40_H_59_N_5_O_7_ + H]^+^ 722.4487, found 722.4492.

***dU-nor-UDC.*** Light yellow syrup, yield 70%; IR: *ν* (cm^−1^) 3441 (O-H), 3016–2862 (C-H), 2255 (C≡C), 1691 (C=O), 1640–1572 (C=C,C=N); ^1^H-NMR: δ = 11.52 (s, 1H), 8.12 (s, 1H), 7.85 (s, 1H), 6.12–6.09 (m, 1H), 5.23 (br, s, 1H), 5.08 (br, s, 1H), 4.42 (br, s, 1H), 4.38–4.18 (m, 3H), 3.85 (br, s, 1H), 3.79 (br, s, 1H), 3.63–3.51 (m, 2H), 3.30–3.19 (m, 2H), 2.78–2.55 (m, 2H), 2.40–2.31 (m, 2H), 2.12–2.02 (m, 2H), 1.98–0.99 (m, 28 H), 0.92 (d, *J* = 6.25 Hz, 3H), 0.83 (s, 3H), 0.55 (s, 3H); ^13^C-NMR: δ = 161.9 (q), 149.3 (q), 146.6 (q), 142.6 (CH), 121.6 (CH), 98.9 (q), 92.9 (q), 87.4 (CH), 84.4 (CH), 72.9 (q), 70.0 (CH), 69.6 (CH), 69.3 (CH), 60.8 (CH_2_), 55.7 (CH), 54.4 (CH), 48.2 (CH_2_), 46.9 (CH_2_), 43.0 (q), 42.9 (CH), 42.0 (CH), 37.6 (CH_2_), 37.1 (CH_2_), 36.1 (CH_2_), 34.7 (CH_2_), 34.2 (CH_2_), 33.6 (q), 33.0 (CH), 32.9 (CH), 30.1 (CH_2_), 28.1 (CH_2_), 28.0 (CH_2_), 27.4 (CH_2_), 26.5 (CH_2_), 24.3 (CH_2_), 23.2 (CH_3_), 20.7 (CH_2_), 18.4 (CH_2_), 18.3 (CH_3_), 11.8 (CH_3_); HRMS calculated for [C_40_H_59_N_5_O_7_ + H]^+^ 722.4487, found 722.4490.

***dA-TUDCA.*** Light yellow syrup, yield 68%; IR: *ν* (cm^−1^) 3340–3284 (O-H), 2918–2860 (C-H), 2240 (C≡C), 1651 (C=O), 1640–1514 (C=C,C=N); ^1^H-NMR: δ = 8.16 (s, 1H), 8.02 (s, 1H), 7.70–7.61 (m, 1H), 7.52 (br, s, 2H), 6.43–6.38 (m, 1H), 5.42–5.25 (m, 2H), 4.48–4.12 (m, 5H), 3.95–3.82 (m, 2H), 3.72–3.59 (m, 1H), 3.55–3.41 (m, 1H), 3.40–3.21 (m, 5H), 3.19–3.12 (m, 2H), 2.85–2.71 (m, 2H), 2.69–2.58 (m, 3H), 2.28–0.99 (m, 27H), 0.93 (s, 3H), 0.88 (d, *J* = 6.41 Hz, 3H), 0.60 (s, 3H); ^13^C-NMR: δ = 155.8 (q), 152.9 (q), 148.2 (q), 146.3 (q), 133.3 (q), 121.7 (CH), 119.2 (CH), 97.4 (q), 88.2 (CH), 85.1 (CH), 72.0 (q), 71.2 (CH), 70.2 (q), 69.6 (CH), 69.3 (CH), 62.1 (CH_2_), 55.6 (CH), 54.3 (CH), 50.1 (CH_2_), 46.9 (CH_2_), 42.9 (q), 42.8 (CH), 42.0 (CH), 40.3 (CH), 39.9 (CH_2_), 39.3 (CH_2_), 38.6 (CH), 37.6 (CH_2_), 37.5 (CH_2_), 37.1 (CH_2_), 36.1 (CH_2_), 34.7 (CH_2_), 33.6 (q), 32.9 (CH_2_), 30.1 (CH_2_), 28.1 (CH_2_), 26.7 (CH_2_), 26.5 (CH_2_), 24.2 (CH_2_), 23.2 (CH_3_), 20.7 (CH_2_), 18.3 (CH_3_), 18.2 (CH_2_), 11.8 (CH_3_); HRMS calculated for [C_44_H_65_N_9_O_8_S + H]^+^ 880.4749, found 880.4751.

***A-TUDCA.*** White yellow syrup, yield 63%; IR: *ν* (cm^−1^) 3340–3311 (O-H), 2929–2870 (C-H), 2243 (C≡C), 1640 (C=O), 1650–1527 (C=C,C=N); ^1^H-NMR: δ = 8.16 (s, 1H), 8.02 (s, 1H), 7.73–7.63 (m, 1H), 7.58 (br, s, 2H), 5.98 (d, *J* = 6.83 Hz, 1H), 5.60–5.55 (m, 1H), 5.43 (d, *J* = 6.25 Hz, 1H), 5.22 (d, *J* = 4.58 Hz, 1H), 5.02–4.97 (m, 1H), 4.48–4.31 (m, 1H), 4.21–4.12 (m, 1H), 3.98–3.87 (m, 2H), 3.72–3.61 (m, 1H), 3.58–3.45(m, 1H), 3.33–3.23 (m, 3H), 2.69–2.55 (m, 3H), 2.15–0.99 (m, 35H), 0.92 (s, 3H), 0.88 (d, *J* = 6.41 Hz, 3H), 0.60 (s, 3H); ^13^C-NMR: δ = 172.6 (q), 156.4 (q), 153.4 (CH), 148.7 (q), 146.6 (q), 134.4 (q), 120.1 (CH), 97.8 (q), 89.7 (CH), 87.0 (CH), 72.0 (q), 71.9 (CH), 71.5 (CH), 70.7 (q), 69.5 (CH), 62.7 (CH_2_), 60.0 (CH), 55.8 (CH), 55.2 (CH), 51.0 (CH_2_), 43.5 (CH), 43.0 (CH), 42.0 (q), 40.0 (CH_2_), 38.8 (CH), 37.7 (CH_2_), 35.9 (CH_2_), 35.6 (CH_2_), 35.4 (CH), 34.6 (CH_2_), 34.2 (q), 33.0 (CH_2_), 32.0 (CH_2_), 28.6 (CH_2_), 28.1 (CH_2_), 27.5 (CH_2_), 27.1 (CH_2_), 25.1 (CH_2_), 23.6 (CH_3_), 21.4 (CH_2_), 18.9 (CH_3_), 18.8 (CH_2_), 18.3 (CH_2_), 12.5 (CH_3_); HRMS calculated for [C_44_H_65_N_9_O_9_S + H]^+^ 896.4698, found 896.4701.

***dG-TUDCA.*** Yield 65%; IR: *ν* (cm^−1^) 3397 (O-H), 2930–2863 (C-H), 2254 (C≡C), 1690, 1646 (C=O), 1638–1552 (C=C,C=N); ^1^H-NMR: δ = 11.18 (s, 1H), 8.02 (s, 1H), 7.79–7.70 (m, 1H), 6.83 (br, s, 2H), 6.25–6.18 (m, 1H), 5.35 (d, *J* = 4.31 Hz, 1H), 4.99 (br, s, 1H), 4.38 (br, s, 1H), 3.92 (d, *J* = 6.42 Hz, 1H), 3.78 (br, s, 1H), 3.63–3.55 (m, 1H), 3.50–3.38 (m, 1H), 3.30–3.20 (m, 4H), 3.05–2.97 (m, 1H), 2.68–2.58 (m, 2H), 2.56–2.49 (m, 4H), 2.12–0.98 (m, 32 H), 0.91 (s, 3H), 0.84 (d, *J* = 6.41 Hz, 3H), 0.59 (s, 3H); ^13^C-NMR: δ = 172.6 (q), 156.5 (q), 154.3 (q), 151.0 (q), 146.7 (q), 132.2 (q), 130.2 (q), 120.3 (CH), 117.2 (q), 95.7 (q), 88.1 (CH), 84.1 (CH), 71.6 (CH), 69.6 (q), 69.5 (CH), 62.5 (CH_2_), 60.1 (CH), 55.7 (CH), 55.1 (CH), 51.1 (CH_2_), 43.5 (CH), 43.03 (CH), 38.8 (CH), 37.7 (CH_2_), 37.4 (CH_2_), 35.9 (CH_2_), 35.5 (CH_2_), 35.3 (CH), 34.6 (CH_2_), 34.2 (q), 33.0 (CH_2_), 32.9 (CH_2_), 32.0 (CH_2_), 28.6 (CH_2_), 28.1 (CH_2_), 27.6 (CH_2_), 27.1 (CH_2_), 25.1 (CH_2_), 23.6 (CH_3_), 22.5 (CH_2_), 21.4 (CH_2_), 18.9 (CH_3_), 18.8 (CH_2_), 12.5 (CH_3_); HRMS calculated for [C_44_H_65_N_9_O_9_S + H]^+^ 896.4698, found 896.4703.

***G-TUDCA.*** Yield 60%; IR: *ν* (cm^−1^) 3329 (O-H), 2933–2867 (C-H), 2254 (C≡C), 1694, 1648 (C=O), 1640–1518 (C=C,C=N); ^1^H-NMR: δ = 11.13 (s, 1H), 8.02 (s, 1H), 7.75 (br, s, 1H), 6.85 (s, 2H), 5.75 (d, *J* = 6.10 Hz, 1H), 5.43 (d, *J* = 6.11 Hz, 1H), 5,11 (d, *J* = 4.89 Hz, 1H), 4.92–4.97 (m, 1H), 4.88 (q, *J* = 5.80 Hz, 1H), 4.38 (br, s, 1H), 4.11 (br, s, 1H), 3.92 (d, *J* = 6.41 Hz, 1H), 3.82 (br, s, 1H), 3.65–3.58 (m, 1H), 3.52–3.38(m, 1H), 3.33–3.23 (m, 4H), 2.66–2.63 (m, 2H), 2.56–2.49 (m, 4H), 2.09–0.98 (m, 30H), 0.92 (s, 3H), 0.86 (d, *J* = 6.41 Hz, 3H), 0.59 (s, 3H); ^13^C-NMR: δ = 172.2 (q), 155.9 (q), 154.1 (q), 150.7 (q), 146.2 (q), 130.1 (q), 129.5 (q) 119.9 (CH), 116.9 (q), 95.1 (q), 88.4 (CH), 85.5 (CH), 71.1 (q), 70.8 (CH), 70.6 (CH), 69.0 (CH), 62.0 (CH_2_), 59.6 (CH), 55.3 (CH), 54.6 (CH), 50.6 (CH_2_), 43.0 (CH), 42.6 (q), 38.3 (CH_2_), 38.2 (CH), 37.3 (CH_2_), 37.2 (CH), 35.42 (CH_2_), 34.9 (CH_2_), 34.1 (q), 33.8 (CH_2_), 32.6 (CH_2_) 32.5 (CH_2_), 31.5 (CH_2_), 28.2 (CH_2_), 27.7 (CH_2_), 27.2 (CH_2_), 26.7 (CH_2_), 24.6 (CH_2_), 23.17 (CH_3_), 20.9 (CH_2_), 18.5 (CH_3_), 18.4 (CH_2_), 12.0 (CH_3_); HRMS calculated for [C_44_H_65_N_9_O_10_S + H]^+^ 912.4647, found 912.4650.

***dU-TUDCA.*** Yield 65%; IR: *ν* (cm^−1^) 3442 (O-H), 3015–2860 (C-H), 1690, 1668 (C=O), 1640–1520 (C=C,C=N); ^1^H-NMR: δ = 8.12 (s, 1H), 8.01 (s, 1H), 7.69–7.61 (m, 2H), 6.32–6.18 (m, 1H), 5.23 (d, *J* = 4.32 Hz, 1H), 5.19–5.11 (m, 1H), 4.42–4.32 (m, 1H), 4.28–4.19 (m, 2H), 3.91 (d, *J* = 6.40 Hz, 1H), 3.78 (br, s, 1H), 3.63–3.55 (m, 2H), 3.50–3.38 (m, 2H), 3.30–3.20 (m, 4H), 3.11–3.02 (m, 2H), 2.65–2.54 (m, 2H), 2.42–2.34 (m, 2H), 2.12–1.08 (m, 29 H), 0.92 (s, 3H), 0.88 (d, *J* = 6.41 Hz, 3H), 0.61 (s, 3H); ^13^C-NMR: δ = 175.4 (q), 172.60 (q), 161.9 (q), 149.3 (q), 146.6 (q), 143.2 (CH), 120.3 (CH), 99.4 (q), 93.5 (q), 88.0 (CH), 85.0 (CH), 80.7 (CH), 71.6 (CH), 69.6 (CH), 61.4 (CH_2_), 60.1 (CH), 55.1 (CH), 51.1 (CH), 43.5 (CH), 43.0 (q), 42.6 (CH_2_), 40.0 (CH_2_), 38.8 (CH), 37.74 (CH_2_), 35.5 (CH_2_), 35.3 (CH), 34.6 (CH_2_), 33.6 (q), 33.8 (CH_2_), 33.0 (CH_2_), 32.9 (CH_2_), 28.6 (CH_2_), 28.1 (CH_2_), 27.7 (CH_2_), 27.1 (CH_2_), 26.7 (CH_2_), 25.1 (CH_3_), 24.6 (CH_2_), 23.6 (CH_2_), 21.4 (CH_2_), 18.9 (CH_3_), 18.8 (CH_2_), 12.4 (CH_3_); MS (ESI, ES+) *m*/*z*: 918 (M + 23).

### 4.8. Cell Lines and Culture

Cell growth inhibition assays were carried out using the leukemia cell line K562 and colon carcinoma HCT116. Cell lines were obtained from ATCC (Manassas, VA, USA) and maintained in RPMI 1640, supplemented with 10% fetal bovine serum (FBS), penicillin (100 Units mL^−1^), streptomycin (100 μg mL^−1^) and glutamine (2 mM) (complete medium); the pH of the medium was 7.2 and the incubation was performed at 37 °C in a 5% CO_2_ atmosphere. Adherent cells were routinely used at 70% of confluence and passaged every 3 days by treatment with 0.05% Trypsin-EDTA (Lonza, Walkersville, MD, USA). K562 cells were routinely fed every 3 days.

### 4.9. Evaluation of Anti-Proliferative Activity (MTT Assay)

The antiproliferative activity of the compounds was tested using the 3-(4,5-dimethylthiozol-2-yl)2,5-diphenyltetrazolium bromide solution (MTT) assay. K562 and HCT116 were seeded in triplicate in 96-well trays respectively at the density of 5 × 10^3^ and 10^4^ in 50 μL of complete medium. Stock solutions (50 mM) of each compound were made in DMSO and diluted in complete medium to give final concentrations of 50, 25 and 10 μM. Untreated cells were placed in every plate as a negative control. The cells were exposed to the compounds, in 100 μL total volume, for 72 h.

### 4.10. Evaluation of Percentage of Apoptosis (Annexin V Staining)

The percentage of apoptotic cells was assessed using the Annexin V assay (Clontech Laboratories, Inc. A Takara Bio Company, Mountain View, CA, USA). Propidium iodide (PI) was used to avoid necrotic cell detection (Annexin−/PI+). The drug-induced apoptotic rate (Annexin+/PI− and Annexin V+/PI+) was compared with the apoptosis in the absence of the drugs used as control (spontaneous apoptosis). K562 cells were cultured in RPMI + 10% FBS in a 6-weels plate for 24 h in the presence of compounds **dA-*nor*-CDC** at concentration of 50 and 25 μM. Cells were washed once with saline buffer (PBS) and resuspended in 250 μL of Binding Buffer 1× containing 100 ng of FITC-labeled annexin V and in control sample, 500 ng of PI. Incubation with annexin V for 15 min on ice in dark was directly followed by flow cytometric analysis of the cells with a FACScan (Becton Dickinson, San Jose, CA, USA) at 488 nm and quantified using the Cell Quest Pro software (Becton Dickinson).

## 5. Conclusions

A conjugation approach by means of click chemistry was exploited in order to synthesize a library of novel fully bio-inspired conjugates combining dA, A, dG, G and dU with CDC, UDC, TUDCA, *nor*-CDC and *nor*-UDC bile acids derivatives. All the nucleoside-BA conjugates were tested for their in vitro anti-proliferative activity against two cancer cells lines and their cytotoxicity towards human fibroblast normal cells. In most of the cases negligible cytotoxicity toward fibroblast was found. Six compounds displayed an interesting anti-proliferative activity with IC_50_ value ≤ 25 μM. In particular, **A-*nor*-CDC** and **dU-*nor*-UDC** were found to be selectively cytotoxic against K562 leukemia cells; **A-CDC** and **G-CDC** were found to be selectively cytotoxic against HCT116; **dA-*nor*-CDC** and **dU-UDC** showed good anti-proliferative activity against both K562 and HCT116. Furthermore, the mechanism of K562 cell death was investigated in the case of **dA-*nor*-CDC** which showed a high percentage of specific apoptosis.

A possible structure–activity relationship was also investigated. In the light of the present data we reason that the cytoselectivity is mainly driven by the nature of the BA but also influenced by the nature of the nucleobase and the sugar form of the nucleoside i.e., deoxy- or ribo-. Therefore, the cytotoxicity could be considered as the result of an interplay of chemical and biochemical properties of the parent biomolecules.

This study confirmed that the conjugation of nucleosides and BAs can actually pave the way to new compounds for anticancer therapy and that unless the structure–activity relationship is not self evident there is a common thread.

## Figures and Tables

**Table 1:** IC_50_ values were determined from the dose–response curves using MTT assay after 72 h incubation time. Results are expressed as the mean of three independent experiments ± SD. Cisplatin was used as a reference compound. Where indicated IC_50_ > 50 inhibition found was <20% at 50 μM; where indicated IC_50_ > 100 inhibition found was <10% at 100 μM.

Molecular Structure	Compound Name		IC_50_ μM	
		K562	HCT116	FIBRO
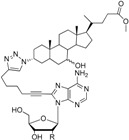	R=H, (7α): **dA-CDC** ^1^	8.5 ± 4.0	>200	>200
	R=H, (7β): **dA-UDC** ^1^	>100	>200	>200
	R=OH, (7α): **A-CDC**	43.5 ± 1.3	23.1 ± 1.7	>100
	R=OH, (7β): **A-UDC**	>50	>50	>100
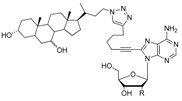	R=H, (7α): **dA-*nor*-CDC**	16.2 ± 2.2	17.0 ± 2.5	91.5 ± 3.5
	R=H, (7β): **dA-*nor*-UDC**	>50	44.8 ± 3.5	>100
	R=OH, (7α): **A-*nor*-CDC**	23.6 ± 1.2	44.1 ± 2.5	>100
	R=OH, (7β): **A-*nor*-UDC**	>50	>50	>100
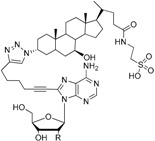	R=H: **dA-TUDCA**	>50	>50	>100
	R=OH, **A-TUDCA**	>50	>50	>100
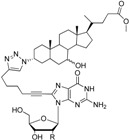	R=H, (7α): **dG-CDC**	>50	>50	>100
	R=H, (7β): **dG-UDC**	>50	>50	>100
	R=OH, (7α): **G-CDC**	>50	25.3 ± 3.8	>100
	R=OH, (7β): **G-UDC**	>50	>50	>100
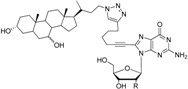	R=H, (7α): **dG-*nor*-CDC**	>50	>50	>100
	R=H, (7β): **dG-*nor*-UDC**	>50	>50	>100
	R=OH, (7α): **G-*nor*-CDC**	>50	>50	>100
	R=OH, (7β): **G-*nor*-UDC**	>50	>50	>100
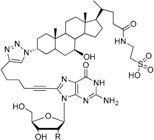	R=H: **dG-TUDCA**	>50	>50	>100
	R=OH, **G-TUDCA**	>50	>50	>100
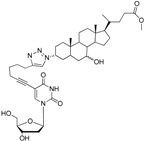	(7α): **dU-CDC**	>50	>50	>100
	(7β): **dU-UDC**	21.5 ± 2.0	23.5 ± 1.6	91.5 ± 4.5
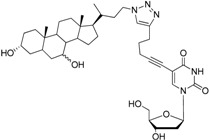	(7α): **dU-*nor*-CDC**	42.9 ± 1.9	>50	>100
	(7β): **dU-*nor*-UDC**	24.8 ± 1.5	43.0 ± 3.5	>100
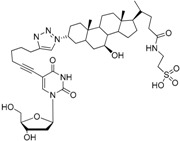	**dU-TUDCA**	>0	>50	>100
	**ALK-dA** ^1^	>50	>50	>100
	**ALK-A**	>50	>50	>100
	**ALK-dG**	>50	>50	>100
	**ALK-G**	>50	>50	>100
	**ALK-dU**	>50	>50	>100
	**3α-N_3_-CDC**	23.0 ± 2.0	31.0 ± 2.3	>100
	**3α-N_3_-UDC**	25.0 ± 1.0	22.0 ± 1.5	>100
	**3α-N_3_-TUDCA**	>50	>50	>100
	**23-N_3_-*nor*-CDC**	21 ± 1.2	25 ± 2.0	79 ± 3.1
	**23-N_3_-*nor*-UDC**	15 ± 1.0	22 ± 1.4	81 ± 2.0
	**CISPLATIN**	5.4 ± 1.0	8.5 ± 1.2	23.6 ± 3.5

^1^ data from ref. [6].
